# Biological Anti-Tumoral Mechanisms of Metformin in Head and Neck Squamous Cell Carcinomas: A Systematic Review

**DOI:** 10.3390/jcm14207258

**Published:** 2025-10-14

**Authors:** Thibaut Buset, Antoine Yanni, Margaux Gerbaux, Cyril Bouland, Xavier Vanden Eynden, Rokneddine Javadian, Jerome R. Lechien, Isabelle Loeb, Edward Boutremans, Sven Saussez, Didier Dequanter

**Affiliations:** 1 Department of Stomatology-Maxillofacial Surgery, CHU-Saint-Pierre, Université Libre de Bruxelles (ULB), 1000 Brussels, Belgium; thibautbuset@gmail.com (T.B.); yanni.antoine@gmail.com (A.Y.); cyril.bouland@ulb.be (C.B.); xavier.vandeneynden@stpierre-bru.be (X.V.E.); rokneddine.javadian@stpierre-bru.be (R.J.); isabelle.loeb@stpierre-bru.be (I.L.); edward.boutremans@stpierre-bru.be (E.B.); 2 Laboratory of Adaptive Immunity, Department of Microbiology, Immunology and Transplantation, KU Leuven, 3000 Leuven, Belgium; margauxgerbaux@gmail.com; 3 Laboratory of Human Anatomy and Experimental Oncology, Faculty of Medicine and Pharmacy, Research Institute for Health Sciences and Technology, University of Mons (UMONS), 7000 Mons, Belgium; jerome.lechien@umons.ac.be (J.R.L.); sven.saussez@umons.ac.be (S.S.); 4 Department of Otorhinolaryngology-Head & Neck Surgery, CHU-Saint-Pierre, Université Libre de Brux-Elles (ULB), 1050 Brussels, Belgium

**Keywords:** head and neck cancer, head and neck squamous cell carcinoma, metformin, anti-tumor effect, biological effect, treatment combination, adjuvant therapy, systematic review

## Abstract

**Background/Objectives**: Recent studies suggest Metformin could be a potential anti-tumoral agent. This review aims to understand the biological anti-tumoral mechanisms of Metformin in head and neck cancer squamous cell carcinomas (HNSCC) both in vitro and in vivo. **Methods:** Two investigators screened publications on the biological anti-tumoral effects of Metformin in HNSCC. The literature search was conducted on PubMed, Cochrane Library, and Scopus using PICOTS and PRISMA statements. **Results:** A total of 30 papers were identified, including 18 studies exploring the effect of Metformin alone and 12 studies exploring its effect in association with another drug or therapy for HNSCC lines. The results suggest that Metformin decreases the proliferation rate of HNSCC through inhibition of cell proliferation by the induction of G0/G1 cell cycle arrest and activation of apoptosis, by regulating proteins involved in carcinogenesis pathways, and also affects the tumor microenvironment by switching the metabolism and activating immune cells. In addition, Metformin can potentiate the efficiency and/or sensibility of other anti-tumoral treatments. The present systematic review highlights the biological anti-tumoral effects of Metformin used alone or in combination with traditional therapies for HNSCC. **Conclusions**: This review of the literature summarizes the biological anti-tumoral effects associated with Metformin alone or in combination with other therapies. While the molecular effects of Metformin on signaling pathways are different when used alone than in combination, they converge in a decreased proliferation of tumor cells and/or a sensitization of HNSCC to other anti-cancer therapies.

## 1. Introduction

Head and neck cancer squamous cell carcinomas (HNSCC) are one of the sixth most common types of cancer diagnosed worldwide [1,2,3,4]. The main therapeutic options for HNSCC include surgery, radiation therapy, and/or chemotherapy. Despite advances in these treatments, the five-year survival rate remains low [5,6,7,8,9]. Current treatments induce many adverse effects with significant long-term toxicity and morbidity. Therefore, alternative, effective, and less toxic therapies should be investigated [10].

The association between diabetes mellitus (DM) and an increased risk of cancer, such as colorectal, breast, uterine, bladder, endometrial, liver, and pancreatic cancers has been demonstrated [11,12]. Recently, Metformin has received attention as a potential anti-cancer agent. Its use in the diabetic population has been associated with decreased cancer incidence and mortality, with limited adverse effects (overall incidence) [13].

Metformin, a biguanide class compound, is the most widely prescribed and well-tolerated drug for type II diabetes [13,14]. Interestingly, diabetic patients treated with Metformin showed a 34% lower incidence of HNSCC [15]. Metformin has many pharmacologic effects. The primary direct mechanism of Metformin’s antineoplastic activity is the activation of AMPK activity, which downregulates the mTOR pathway. It also regulates phosphoinositide 3 kinase (PI3K) and mammalian target of rapamycin complex 1 (mTORC1) [16,17,18,19,20,21]. Moreover, by downregulating the mTOR pathway, Metformin acts as a negative regulator of the Warburg effect, a common metabolic characteristic of malignant cells for proliferation and resistance to drugs [14,22,23,24]. Metformin also has a direct effect on cell cycling and apoptosis [14].

Since the interactions between cancer and several biological mechanisms of Metformin have been documented, it is important to understand the molecular mechanisms of Metformin in cancer prevention and therapy, especially in HNSCC, which has limited therapy strategies and poor prognosis.

After conducting a literature review focused on the preventive and therapeutic effects of Metformin [25], the authors conducted a second literature review to describe the biological mechanisms that could explain these effects described in the literature. The present study is the first to review the biological mechanisms of the anti-tumoral effects of Metformin on HNSCC demonstrated in both in vitro and in vivo studies.

## 2. Materials and Methods

### 2.1. Type of Studies

The present systematic review included studies that assessed the biological effects of Metformin, either alone or in combination with other therapies, on HNSCC. We collected experimental studies, including both in vitro and in vivo assays, concerning the use of Metformin in HNSCC. All tumoral sites of HNSCC were included, except nasopharyngeal tumors, as they present different cancer etiology, epidemiology, and therapeutic options.

### 2.2. Exclusion Criteria

Studies were excluded for the following reasons: (1) studies that did not research the effect of Metformin in cancer cell therapy; (2) studies that did not investigate the impact of Metformin on HNSCC treatment; (3) reviews, personal opinions, letters, conference abstracts, and book chapters; (4) full text not available or not written in English, public communications or posters.

### 2.3. Outcomes

The aim of the study was to define, based on preclinical data, the biological mechanisms explaining the preventive and therapeutic effects of Metformin in head and neck cancers. The results are presented in four categories: in vitro studies with Metformin as the sole treatment, in vivo studies with Metformin as the sole treatment, in vitro studies with Metformin as an adjuvant therapy, and in vivo studies with Metformin as an adjuvant therapy.

### 2.4. Search Strategy

Two independent authors (B.T. and Y.A.) searched on PubMed, Cochrane Library, and Scopus to identify articles published between 1 January 2010, and 1 January 2023, evaluating the biological anti-tumoral mechanisms of Metformin in HNSCC. The following search terms were used: “Metformin” and “head and neck squamous cell carcinoma”, and were employed in every possible spelling, as well as synonyms, acronyms, and key or text words. The Boolean operator used was AND. The PubMed search included Mesh terms such as Mouth Neoplasms OR Squamous Cell Carcinoma of Head and Neck OR Head and Neck Neoplasms OR Carcinoma, Squamous Cell AND Metformin.

### 2.5. Study Selection

The criteria for consideration of study inclusion were based on the Population, Intervention, Comparison, Outcome, Timing, and Setting (PICOTS) framework [26]. Three authors (B.T., Y.A., and B.C.) independently reviewed and extracted data from studies, adhering to the PRISMA checklist for systematic reviews [27]. The three authors analyzed the full texts of selected studies. The search strategy results were reviewed for relevance, and the reference lists of these publications were examined for additional pertinent studies. There were no discrepancies in synthesized data among the three authors.

### 2.6. Data Collection Process and Data Items

One principal author (B.T.) collected all the required information from the selected articles, including authors’ names, years of publication, country, study design, assays performed, cell line or in vivo model on animals used, treatment used, results, and conclusions. A second author (Y.A.) verified the collected data.

## 3. Results

### 3.1. Article Selection

**Figure 1:**
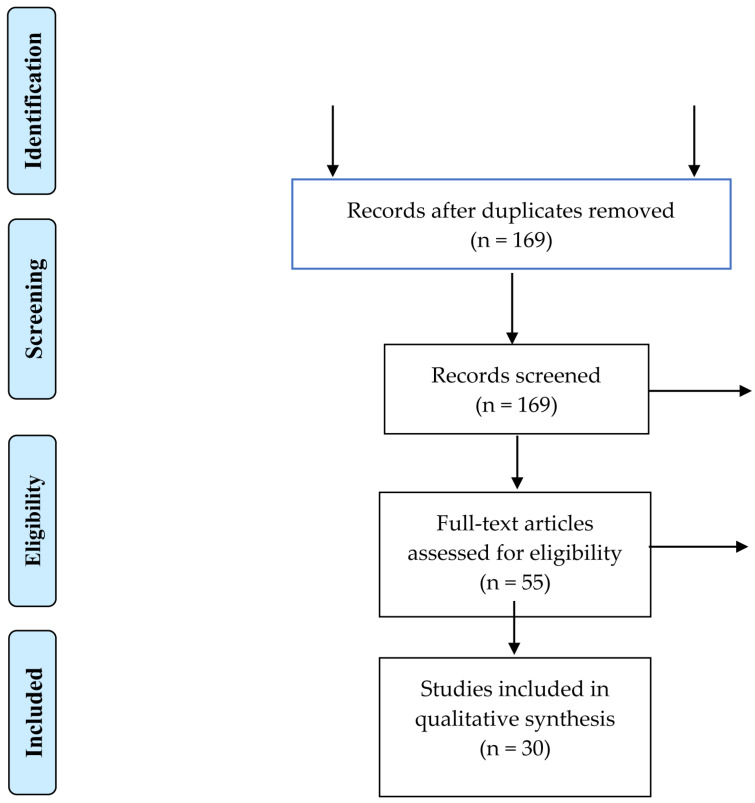
Prisma flow diagram.

After the initial selection step, 169 articles were identified, and 114 articles were excluded based on critical reading. Following the revision process and a full-text review, 25 papers did not meet the inclusion criteria, as illustrated in the PRISMA diagram flowchart (Figure 1). Ultimately, 30 articles published in English between January 2012 and January 2023 were selected. The studies represented various world regions: China (*n* = 14), United States (*n* = 8), Taiwan (*n* = 2), Canada (*n*= 1), Korea (*n* = 1), India (*n* = 1), Brazil (*n* = 1), Japan (*n* = 1), and Saudi Arabia (*n* = 1). The majority of the studies (*n* =18) explored the effect of Metformin alone in the treatment of HNSCC, with eight studies conducting in vitro assays (Table 1) and ten performing in vivo assays (Table 2). Additionally, 12 studies evaluated the effect of Metformin in combination with another therapy, with three studies conducting in vitro assays (Table 3), while nine studies were performed in vivo (Table 4).

### 3.2. Metformin Used Alone

**Figure 2:**
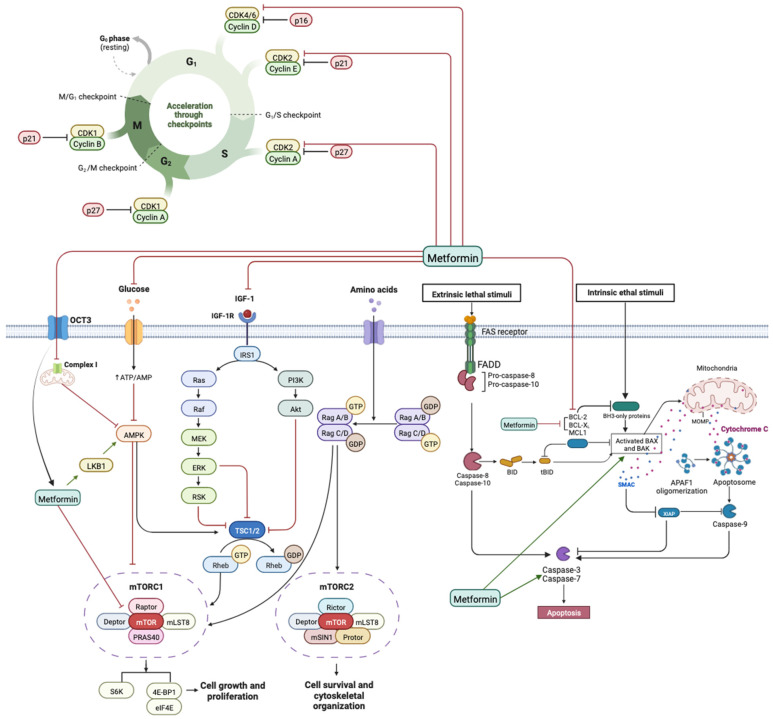
Effects of Metformin on cell cycle regulation in cancer, the mammalian target of rapamycin (mTOR) signaling pathway, and its role in apoptosis through extrinsic and intrinsic pathways. By BioRender.com (2022). Retrieved from https://app.biorender.com/biorender-templates, accessed on 1 June 2022.

All studies (*n* = 18) examining the biological mechanisms of Metformin alone reported a decrease in the proliferation rate of head and neck squamous cell carcinoma (HNSCC) (Table 1 and Table 2) [28,29,30,31,32,33,34,35,36,37,38,39,40,41,42,43,44]. Among the eight in vitro studies [30,33,34,35,36,37,39,40,41,42] (Table 1), the main mechanisms of action attributed to Metformin in HNSCC included inhibition of cell proliferation and induction of apoptosis [12,31,35,36,42]. Several molecular pathways were involved in these effects. Notably, the inhibition of the mTOR signaling pathway was highlighted in multiple studies [36,38,41] (Figure 2). This inhibition was shown to be linked to the expression of organic cation transporter 3 (OCT-3), which facilitates Metformin uptake and mediates its effect on mTOR signaling [35]. Conversely, in studies where OCT-3 was downregulated, Metformin failed to activate AMP-activated protein kinase (AMPK) and subsequently did not inhibit the mTORC1 pathway [38], underlining the importance of OCT-3 in Metformin responsiveness.

**Table 1:** In vitro studies exploring the effect of Metformin alone.

Authors, Year, Country	Study Design	Assays	Cell Line	Treatment +/− Control	Concentration/IC50 or Effective Dose	In Vivo Toxicity/Side Effects	Results
Wang et al., 2020 [12], China	In vitro	Cell proliferation assay/Cell cycle assay/Cell apoptosis assay/WB/Construction of YAP-overexpressing cell lines/RT-q PCR analysis	CAL27 and SCC25	Metformin	Not specified No IC_50_ reported	Not reported	Metformin promotes cell apoptosis, inhibits cell proliferation, and stimulates the Hippo signaling pathway in OSCC cells. Metformin inhibits OSCC cell growth by decreasing YAP and decreases mTOR and c-Myc through the downregulation of YAP
Wei et al., 2021 [41], China	In vitro	Cell culture and treatment/Plasmid construction and lentiviral transfection/Cell proliferation assay/Colony formation assay/Cell cycle analysis/qRT-PCR/Immunoprecipitation and WB/IF staining/Chromatin immunoprecipitation assays	SCC-15	Metformin	Not specified No IC_50_ reported	Not reported	Metformin inhibits OSCC cell proliferation by suppressing NGFR proteolysis, which promotes OSCC cell proliferation.
Patil et al., 2020 [40], Saudi Arabia	In vitro	Preparation of the single-cell suspension/MTT assay for cell viability/RT-PCR for stemness related transcription factors/Flow cytometry analysis for CD44 expression	OSCC	Metformin	Not specified No IC_50_ reported	Not reported	Metformin showed downregulation in the gene expressions of stemness related transcription factors OCT4, SOX2, NANOG, c-Myc, and KLF4 in a dose-dependent as well as time-dependent manner.
Zhang et al., 2019 [30], China	In vitro	Cell isolation and culture/Indirect co-culture system/Cell count and viability detected by Muse^®^ count and viability assay kit/Apoptosis detection by flow cytometry/Mitochondrial membrane potential measurement/Assay for ROS production/ATP and l-lactate production measurement/WB	Normal oral fibroblasts (NOFs), OSCC	Metformin	Metformin used at **0–10 mM** in vitro; **IC_50_ ≈ 2.992 mM** in OSCC cells.	Not reported	Metformin inhibits cell growth and induces apoptosis in human OSCC cells in a dose-dependent manner. Co-culture with NOFs, Metformin induces metabolic reprogramming and autophagy in OSCC
Sun et al., 2016 [33], China	in vitro	Cell culture/Cell proliferation assay/Real time qRT–PCR/WB/Statistical analyses	FaDu	Metformin	25–125 mM Metformin; dose- and time-dependent inhibition of FaDu cell proliferation; no IC_50_ reported.	Not reported	Metformin inhibited FaDu cell proliferation in a dose and time-dependent manner, downregulated miR-21-5p, and upregulated PDCD4 mRNA and protein expression
Guimaraes et al., 2016 [34], Brazil	in vitro	Cell culture and hypoxia/Drug sensitivity assay and groups/RNA isolation and qRT-PCR/RNA isolation and qRT-PCR/WB/IHC/Proliferation assay/Wound scratch assay/DNA fragmentation assay/Bioinformatics and interaction network analysis/In-cell Western	SCC9 cells HaCaT cells	Metformin	Metformin at **10, 20, and 50 µg/mL** for 24 h in vitro; **20 µg/mL** chosen for key experiments; No IC_50_ reported.	Not reported	Metformin favored an increase in PDH levels in hypoxic conditions, reduced HIF-1α mRNA and protein levels, inhibited migration, increased the number of apoptotic cells and increased the transcription of caspase 3
Patel et al., 2013 [37], USA	in vitro	Cell culture and antibodies/IHC/RNA interference/Western blotting/Cell viability	Human oral dysplastic and HNSCC	Metformin, vehicle control	Metformin at **3 mM** applied for **72 h** in vitro No **IC_50_** reported	Not reported	The inhibition of OCT-3 expression and activity in HNSCC cells prevented Metformin-induced AMPK- activation and mTORC1 pathway inhibition
Sikka et al., 2012 [39], USA	in vitro	Cell culture and treatments/Cell viability assay/WB/Cycloheximide experiment to determine protein stability	FaDU and Detroit 562	Metformin, plain medium (control)	5–20 mM Metformin applied in vitro (24–72 h); produced dose-dependent growth inhibition (18–81% depending on time and cell line). No IC_50_ reported.	Not reported	Treatment with Metformin inhibited the growth of HNSCC caused by G1 arrest leading to a decrease in Cdks (2, 4 and 6), cyclins (D1 and E) and Cdk inhibitors (p15, p16, p18 and p27) and decreased the levels of oncogenic proteins Skp2 and β-Trcp

Abbreviations: IHC: Immunohistochemistry; IF: Immunofluorescence; ATP: adenosine triphosphate; ROS: reactive oxygen stress; WB: Western blot; qRT-PCR: Real-Time Quantitative Reverse Transcription PCR; OSCC: Oral Squamous Cell Carcinoma; NOFs: Normal oral fibroblasts; HNSCC: Head and neck squamous cell carcinoma; PDCD4: Programmed Cell Death 4; PDH: Pyruvate Dehydrogenase; HIF-1α: Hypoxia-inducible factor 1-alpha; mRNA: messenger ribonucleic acid; mTOR: mammalian target of rapamycin; AMPK: 5′ AMP-activated protein kinase; mTORC1: mammalian target of rapamycin complex 1; G1: Gap 1 phase; Cdks: cyclin-dependent kinases; Skp2: S-Phase Kinase Associated Protein 2; β-Trcp: Beta-transducin repeats-containing proteins; YAP: Yes-associated protein; NGFR: nerve growth factor receptor; OCT4: octamer-binding transcription factor 4; SOX2: SRY-Box Transcription Factor 2; KLF4: Kruppel-like factor 4.

**Table 2:** In vitro and in vivo studies exploring effect of Metformin alone.

Authors, Year, Country	Study Design	Assays	Cell Line	Treatment +/− Control	Concentration/IC_50_ or Effective Dose	In Vivo Toxicity/Side Effects	Results
Wu, Yeerna et al., 2019 [28], China	In vitro in vivo	Vector construction and Lentiviral infection using Gateway system/WB/RNA isolation, qPCR analysis, gene expression profiling, and GSEA analysis/Seahorse assay for O_2_ consumption/IF and IHC	CAL27, CAL33, and UMSCC47	HNSCC lines CAL 27, CAL 33 and UMSCC 47 Nude mice Xenograft tumor Metformin 2.5 mg/mL or water	In vitro: Metformin at 3 mM (24 h). In vivo: 2.5 mg/mL in drinking water (~2 µg/mL plasma).	No observed toxic effects: body weight, serum glucose, and insulin remained unchanged; no adverse signs reported.	User Metformin activates AMPK and inhibits mTOR by targeting complex I in HNSCC cells, inhibits mitochondrial complex I activity in HNSCC cells and causes the association of dephosphorylated 4E-BP1 with eIF4E and the disruption of the association between eIF4E and eIF4G
Wu, Tang et al., 2019 [29], China	In vitro in vivo	Cell infection and transfection to generate lentivirus expressing shDNMT1/RNA extraction and RT-qPCR/LncRNA Microarray analysis/WB/Proliferation assay/Annexin-V/PI Double staining for apoptosis analysis/Tumor xenograft in nude mice/RNA RIP/MSP/SAHH activity assay	FaDu cells	Exposed to different concentrations (0, 2, 4, 6, 8 mM 48 h) or at 8 mM various hours (0, 12, 24, 36, 48, 72) Tumor xenograft in nude mice	Metformin: 0–8 mM, 48 h dose-response; 8 mM across 0–72 h time-response. Inhibited FaDu cell proliferation and induced apoptosis via SAHH/DNMT1/SNHG7 axis.	No toxicity or side-effect data reported. Xenograft experiments showed tumor suppression, but animal welfare outcomes are not described.	Metformin suppresses lncRNA SNHG7, inducing an inhibition of FaDu cell proliferation in a time- and dose-dependent manner. Metformin also sensitizes FaDu cells to radiotherapy and taxol effects through decreasing lncRNA SNHG7.
Verma et al., 2018 [31], USA	In vitro in vivo	Photoacoustic imaging with co-registered ultrasound/Fluorescence imaging/Magnetic resonance imaging/IHC	FaDu	Metformin, water (control)	200 mg/kg Metformin, intraperitoneal, daily for 5 days. Increased tumor %sO_2_ (≈50→62%) and HbT levels.	No weight loss or toxicity noted. No hemodynamic alterations in salivary glands, indicating safety.	Metformin therapy is associated with an increase in %sO_2_ (oxygen saturation) and HbT (hemoglobin total) levels in treated tumors.
Tassone et al., 2018 [32], Korea	In vitro in vivo	Cell Lines and culture/Flow cytometry/CAV1 knockdown/Immunohistochemistry/TUNEL Assay/IHC	CAL27, BJ1	Metformin	Oral Metformin reduced tumor size by ~45%, lowered MCT1 expression by ~28%, and increased apoptosis by ~1.8× in coinjection xenografts. Exact dose not reported.	Not reported; no mention of adverse effects or toxicity measurements in the study	Metformin decreased the size of the tumor by 45%, reduced MCT1 staining and increased carcinoma cell apoptosis 1.8-fold
Chen et al., 2017 [42], Taiwan	In vitro in vivo	Human oral cancer tissues and IHC/Cell culture/Immunoblot analysis/Indirect IF and time lapse microscopy/RNA extraction, and quantitative RT-PCR/Cell viability assay and colony formation assay and flow cytometry analysis of the cell cycle/ChIP/Migration, invasion, and wound-healing assays/Animal experiments and IHC	SAS and SCC25 and Cal27	Metformin or PBS (control)	In vitro: 10 mM Metformin inhibited SAS, CAL-27, and SCC25 cell proliferation and invasion. In vivo: 5 mM Metformin (in drinking water) reduced tumor growth in xenograft models.	No significant toxicity observed—body weight remained stable; no adverse events were reported.	Metformin inhibited cancer development, such as the growth and metastasis of oral cancer cells, in part through LSF/Aurora-A signaling.
Luo et al., 2012 [36], China	In vitro in vivo	Cell culture/Cell proliferation assay/Cell clonogenic assay/Cell cycle and apoptosis analysis/WB/In vivo anti-tumor activity/TUNEL(Terminal deoxynucleotidyl transferase-mediated nick end labeling staining)/IHC	CAL27, WSU-HN6 and SCC25	Metformin, water (control)	In vitro: 5–20 mM inhibits proliferation, >90% reduction in colony formation at 20 mM; apoptotic rates significantly increased at 48 h.	Oral administration (200 µg/mL via drinking water) inhibited tumor growth with no observed toxicity or weight loss in mice.	Metformin inhibited OSCC cell proliferation in a time- and dose-dependent manner. It induced apoptosis in OSCC cells by down-regulating the anti-apoptotic proteins Bcl-2 and Bcl-xL and up-regulating the pro-apoptotic protein Bax. Metformin activated AMPK and decreased mTOR and S6 kinase, leading to a decrease in cyclin D1, CDK4, and CDK6 protein levels and phosphorylation of the retinoblastoma protein.
Vitale-Cross et al., 2012 [38], China	In vitro in vivo	Reagents, cell lines, tissue culture, and transfections/Western blotting, cell proliferation and viability assays, and ATP assay/Experimental animal model and plasma levels of IGF-1 and insulin/IHC and IF/T-cell proliferation assay and flow cytometry	Cal-27, HN12, HN13, and Hep2 and HeLa cells	Metformin, sterile saline (control)	In vitro: 10–20 mM Metformin significantly inhibited proliferation and induced apoptosis in HN12, HN13, and Hep2 cells.	No significant toxicity or adverse effects were reported in mice treated with Metformin. Tumor growth was significantly inhibited	Treatment with Metformin inhibited HNSCC cell proliferation, downregulated the mTORC1 pathway activity, and reduced the size and progression of premalignant lesions.
Madera et al., 2015 [35], USA	In vitro in vivo	Reagents, cell lines, and tissue culture/Lentiviral constructs for OCT3 knockdown/WB, cell proliferation, and colony formation/Xenograft HNSCC tumor models/Histologic studies and IHC	CAL27 (ATCC), CAL33, and UMSCC47	Metformin, water (control)	In vitro: 0–30 mM Metformin inhibited proliferation and colony formation; IC_50_ not specified. In vivo: 2.5 mg/mL in drinking water resulted in plasma concentrations of approximately 2 µg/mL.	No significant toxicity or adverse effects were reported; tumor growth was significantly inhibited.	Metformin inhibits mTOR signaling and tumor growth in HNSCC cells that requires the expression of organic cation transporter 3 (OCT3/SLC22A3), a Metformin uptake transporter
Curry et al. 2017 [43], USA	In vivo	IHC, TUNEL apoptosis assay		Metformin	In vivo: 500 mg/day, increased to 1000 mg twice daily over 6 days, administered for 9+ days before surgery	No significant adverse effects reported. Metformin was well tolerated.	Metformin modulates metabolism in the HNSCC microenvironment, through the increase in reduced catabolism and senescence markers in stromal cells as well as carcinoma cell apoptosis
Curry et al. 2018 [44], USA	In vivo	IHC, TUNEL apoptosis assay		Metformin	Metformin was initiated at a dose of **500 mg/day** and increased to **1000 mg twice daily** by day 6 of the treatment course. The total treatment duration was **9 or more days** prior to surgical resection.	• **Tolerability**: The study reported that Metformin was well tolerated by patients during the treatment period. The average treatment course was **13.6 days**. • **Adverse effects**: No significant adverse effects or toxicity were noted in the study, suggesting a favorable safety profile at the administered doses	Metformin alters the immune tumor microenvironment with an increased infiltrate of CD8+ Teff and FoxP3 Tregs at the invasive tumor margin of lymph nodes with extra-capsular extension

Abbreviations: IHC: Immunohistochemistry; WB: Western blot; IF: Immunofluorescence; qPCR: Real-time quantitative polymerase-chain-reaction; GSEA: Gene Set Enrichment Analysis; shDNMT1: 1 shRNA targeting DNMT1; RT-qPCR: Quantitative reverse transcription quantitative polymerase-chain-reaction; lncRNA: Long noncoding RNAs; RIP: Immunoprecipitation; MSP: Methylation Specific PCR; SAHH: S-adenosylhomocysteine hydrolase; mTOR: mechanistic target of rapamycin; RT-PCR: Reverse transcription polymerase chain reaction; ChIP: Chromatin immunoprecipitation; TUNEL: Terminal deoxynucleotidyl transferase dUTP nick end labeling; AMPK: 5′ AMP-activated protein kinase; mTOR: mammalian target of rapamycin; HNSCC: Head and neck squamous cell carcinoma; 4E-BP1: Eukaryotic translation initiation factor 4E; eIF4E: Eukaryotic Translation Initiation Factor 4E; eIF4G: Eukaryotic translation initiation factor 4 G; SNHG7: Small Nucleolar RNA Host Gene 7; sO2: saturation of oxygen; IGF-1: insulin-like growth factor; ERK1/2: extracellular signal regulated protein kinase; MCT1: Monocarboxylate transporter 1; LSF: Late SV40 Factor; OSCC: Oral Squamous Cell Carcinoma; Bcl-2: B-cell lymphoma 2; Bcl-xL: B-cell lymphoma-extra-large; CDK: cyclin-dependent kinase; PBS: Phosphate-buffered saline; TUNEL: terminal deoxynucleotidyl transferase dUTP nick end labeling.

Additionally, Metformin was reported to suppress the proteolytic cleavage of nerve growth factor receptor (NGFR), a transmembrane protein implicated in cancer cell proliferation and metastasis, thereby contributing to apoptosis in HNSCC cells [12]. Another proposed mechanism involved the reduction in Yes-associated protein (YAP), a known oncogenic effector, which was associated with downstream inhibition of c-Myc and mTOR, ultimately leading to decreased cell proliferation and increased apoptosis [42].

Ten in vivo studies investigating the biological effects of Metformin alone supported its anti-tumoral properties (Table 2) [28,29,31,32,36,38,43,44,45]. Metformin has also been shown to exert antitumor activity in thyroid cancers, suggesting its potential role as an adjuvant therapeutic agent [46,47,48]. Metformin was shown to activate AMPK and inhibit mTOR signaling by targeting mitochondrial complex I in HNSCC cells [29]. Furthermore, Wu et al. [29] reported that Metformin could suppress the expression of the long noncoding RNA SNHG7 through the activation of S-adenosylhomocysteine hydrolase (SAHH), contributing to inhibited proliferation in FaDu cell-derived tumors.

In a study by Tassone et al. [32], treatment with Metformin led to a 45% reduction in tumor volume, a 1.8-fold increase in apoptotic cells, and a 28% decrease in monocarboxylate transporter 1 (MCT1) expression—an importer of lactate associated with the reverse Warburg effect. While these findings suggest altered tumor metabolism, the authors noted that further studies are required to establish direct causality between observed transporter expression changes and metabolic reprogramming. Similarly, Zhang et al. [30] reported increased hemoglobin oxygen saturation in Metformin-treated tumors, suggesting a possible effect on the tumor microenvironment, although no direct mechanistic link was confirmed.

Chen et al. [43] identified the inhibition by Metformin of LSF/Aurora-A signaling, a pathway known to be involved in tumor cell proliferation and mitotic regulation in HNSCC. Other studies reported reduced levels of S6 kinase, cyclin D1, CDK4, and CDK6, as well as a downregulation of anti-apoptotic proteins Bcl-2 and Bcl-xL and upregulation of the pro-apoptotic protein Bax [36]. These molecular alterations were associated with reduced tumor progression and lesion size [38].

Curry et al. [44] also described changes in the HNSCC tumor microenvironment upon Metformin treatment, including increased markers of senescence and catabolism in stromal cells, as well as increased apoptosis in carcinoma cells. While suggestive of metabolic modulation, these findings were associative and require further functional validation. In addition, Metformin was reported to influence immune cell infiltration, with an increased presence of CD8+ effector T cells and FoxP3+ regulatory T cells at the invasive tumor margin of lymph nodes with extracapsular extension [45], suggesting potential immune modulation, although the functional consequences of this altered immune profile remain to be fully elucidated.

### 3.3. Metformin in Combination

Among the 30 selected studies, 12 investigated the combinatory effects of Metformin with another therapeutic agent, demonstrating an adjuvant or synergistic action (Table 3 and Table 4) [9,20,24,49,50,51,52,53,54,55,56]. However, most of these studies lacked detailed quantitative comparisons between the effects of Metformin alone, the second drug alone, and the combination. Such data are essential for evaluating whether the observed effects are additive, synergistic, or merely overlapping.

**Table 3:** In vitro studies exploring the effects of Metformin as an adjuvant therapy.

Authors, Year, Country	Study Design	Molecules	Assay	Cell Line	Concentration/IC_50_ or Effective Dose	In Vivo Toxicity/Side Effects	Treatment	Results
Lindsay et al., 2019 [57], Canada	in vitro	Metformin curcumin	Cell culture and drug treatment protocol/Cell proliferation assay/Whole cell lysate and WB/WB quantification and analysis/RNA extraction, purification, and droplet digital PCR/IF	CAL-27, SCC-90, SCC-152, SSC-6	Not specified; study reported efficacy in proliferation reduction and apoptosis induction, but no dose or IC_50_ details. Combination did not show synergy in HNSCC cell lines.	Not reported	Metformin + curcumin	Metformin induced apoptosis in HPV+ cell lines and slowed the rate of proliferation.
Kuo et al., 2019 [49], USA	in vitro	Metformin cisplatin	Cell Lines and cultures/FACS identification of ALDH+ and ALDH- cell populations/Cell proliferation assay/TUNEL assay/WB/qRT-PCR and siRNA knockdown/IF/Computational prediction of metformin binding energy	JLO-1 and HN30	HN-30: 8–12 mM Metformin, then 1–20 μM cisplatin. JLO-1: 0.5–0.7 mM Metformin, then cisplatin. Metformin prevented cisplatin cytotoxicity	Not reported	Metformin and cisplatin	Metformin protected HNSCC against cisplatin therapy in vitro.
Yang et al., 2019 [20], USA	in vitro	BPTES/Metformin	WB/Cell viability assay/Cells were stained with necrosis and apoptosis dyes/Flow cytometry analysis/Crystal violet staining/MTT assay and WB	FaDu and Detroit 562	BPTES: 1–20 µM (dose-dependent inhibition); Metformin: 10 mM (effective in suppressing growth); Combination: additive effects on viability and apoptosis	Not reported	BPTES and Metformin PBS (control)	BPTES and Metformin inhibits the cell growth of HNSCC cells and induce G1-phase arrest due to the decrease in the expression of CDK1/cyclin B1 and Cyclin E2.

Abbreviations: WB: Western blot; PCR: Polymerase chain reaction; FACS: Fluorescence-activated cell sorting; ALDH: Aldehyde dehydrogenases; TUNEL: Terminal deoxynucleotidyl transferase dUTP nick end labeling; qRT-PCR: Real-Time Quantitative Reverse Transcription PCR; siRNA: Small interfering RNA; MTT: 3-(4,5-dimethylthiazol-2-yl)-2,5-diphenyltetrazolium bromide; HPV: Human Papilloma Virus; HNSCC: Head and neck squamous cell carcinoma; BPTES: (bis-2-(5-phenylacetamido-1,2,4-thiadiazol-2-yl)ethyl; CDK: cyclin-dependent kinase saturation of oxygen; CDK: cyclin-dependent kinase; PBS: Phosphate-buffered saline.

**Table 4:** In vitro and in vivo studies exploring the effects of Metformin as an adjuvant therapy.

Author, Year, Country	Study Design	Molecules	Assay	Cell Line	Concentration/IC_50_ or Effective Dose	In Vivo Toxicity/Side Effects	Treatment	Results
Chen et al., 2021 [55], China	In vitro in vivo	Metformin/ C1632	Cell culture/MTT assay/WB/Wound healing and Transwell assay/Xenograft mouse experiment	SCC9, CAL27	Metformin: 5–20 mM; C1632: 1–10 μM, showing synergistic inhibition of OSCC cell migration and proliferation.	No significant toxicity reported in mouse xenograft models; treated animals maintained stable weight and health.	Metformin and C1632	C1632 and Metformin synergistically downregulated the expression of LIN28 and inhibited the migration capacity of OSCC cell lines. These synergistic effects were exerted via the AMPK-dependent pathway.
Hu et al., 2020 [58], China	In vitro in vivo	Metformin/CDK4/6 inhibitors	Cell viability assay and synergy analysis/Colony formation assay/Cell cycle analysis/WB/Immunostaining/Senescence-associated β-galactosidase staining/qRT-PCR/Antibody array/Sphere-forming assay/Flow cytometry analysis/ELISA assay/Xenograft mouse experiment	CAL27, HSC3, HSC6 and MCF7	Metformin: 5–20 mM; LY2835219: **0.1 µM**, **0.3 µM**, **1.25 µM** depending on cell line. Synergistic inhibition of HNSCC cell proliferation and cell cycle progression.	No significant toxicity or weight loss observed in xenograft models; treatment was well tolerated	Metformin and LY2835219 (a CDK4/6 inhibitor)	Both molecules show synergistic effects on HNSCC in vitro and in vivo. They also synergistically promote cell cycle arrest. By inhibiting the mTOR and stat3 pathways, Metformin modulates the profiles of the SASP induced by the CDK4/6 inhibitor. Metformin blocks the SASP-induced stemness caused by a CDK4/6 inhibitor, and the blockade of the IL6-stat3 axis by Metformin is associated with stemness inhibition.
He et al., 2019 [9], China	in vitro in vivo	Metformin and 4SC-202	Cell proliferation assay/IHC/Colony formation assay/Cellular apoptosis assay/TUNEL/WB/Cell transfection/qRT-PCR/Co-immunoprecipitation	HSC6 and HSC3	**Metformin: ~16 mM** **4SC-202 at ~0.4 µM** in combination with Metformin ~16 mM. PMC In vivo: In the xenograft model: Metformin at **100 mg/kg** and 4SC-202 at **80 mg/kg**.	No significant toxicity or weight loss observed in animal models; treatment well tolerated.	Metformin and 4SC-202	Metformin and 4SC-202 induced intrinsic apoptosis and suppressed the proliferation of the cells;
Yin et al., 2019 [50], China	in vitro in vivo	Gefitinib/Metformin	Transwell migration assay/RNA extraction and RT-qPCR analysis/IF/WB/Histopathological and IHC/CCK-8 assay analyses/Colony formation assay/EdU incorporation assay/Cytokine antibody array/ELISA/Flow cytometry/Target gene expression knockdown/GSEA	CAL27, JHU011, FaDu and SCC9	Metformin: 5 mM; Gefitinib: 1–10 μM; combination sensitizes cells by modulating TAM-related pathways.	No significant toxicity or weight loss observed in animal models; treatment was well tolerated	Gefitinib/Metformin or PBS (control)	Metformin sensitized HNSCC cells to gefitinib through the inhibition of CCL15 expression in M2-type TAMs and the suppression of CCR1 surface expression. These pathways are associated with resistance to gefitinib.
Yin et al., 2018 [51], China	in vitro in vivo	Metformin/gefitinib	Cell culture/Flow cytometry/RT-qPCR/WB/histopathological analysis and IHC/Cell viability assay/apoptosis detection/Cell cycle analysis	CAL27, HSC3, and SCC4	Metformin: 100 µM; Gefitinib: 1 µM combination enhanced apoptosis and cell cycle arrest.	No significant toxicity or weight loss observed; treatment was well tolerated in animal models	PBS, gefitinib, and Metformin	Metformin sensitized to gefitinib treatment both in vivo and in vitro.
Siddappa et al., 2017 [52], India	in vitro in vivo	Metformin/curcumin	Establishment of animal model/Histology and IHC/Expression profiling/Chemoprevention study/Clinical response evaluation/Primary culture and expression profiling/FACS assay/Cell migration assays/Clonogenic survival assay	4NQO until development of dysplastic oral potentially malignant lesions	Not reported	No reported toxicity or adverse effects; treatment was well tolerated and improved survival in animals	Metformin and curcumin	Chemopreventive treatment significantly decreased the tumor volume compared to controls and improved overall survival in animals. Additionally, it showed a concordant and consistent downregulation of the CSC markers following combination treatment.
Harada et al., 2016 [24], Japan	In vitro in vivo	Metformin and 5FU	Cell lines and cell culture/In vitro cell growth assay/TUNEL assay/Lactate colorimetric assay/WB analysis/Nude mice and tumor inoculations/In vivo treatment protocol/IHC.	HSC2, HSC3 and HSC4	**In vitro**: Metformin at **4 mg/mL** 5-FU at **2.5 μg/mL** In vivo: Metformin: **200 mg/kg** (intraperitoneal, i.p.) in mice. 5-FU: **10 mg/kg** (i.p.) in mice	Well tolerated, no significant toxicity or weight loss; no adverse effects observed in mice	5-FU and Metformin	Metformin in combination with 5-FU inhibited cell growth and induced apoptosis in OSCC cell lines. This combination downregulated HIF-1α and mTOR expression while upregulating AMPKα. The combined treatment was more effective in reducing tumor growth compared to Metformin or 5-FU alone.
Qi et al., 2016 [53], China	In vitro In vivo	Metformin/cisplatin	Cell line and cell culture/MTT assay/WB/Plasmid transfections/Luciferase assay/IF/HIF-1α knockdown with siRNA/qRT-PCR/Flow cytometric analysis/Nude mice and tumor inoculations/TUNEL assay/IHC	TCA8113, HSC3 and SCC3	In vitro: Metformin: **10 μM** In vivo: Metformin was 10 mg/kg (oral) in the Qi et al. study, not 250 mg/kg/day. Cisplatin dose used was 10 mg/kg	Well tolerated, no significant toxicity or weight loss observed; enhanced tumor apoptosis without damage to normal tissues	Metformin/cisplatin	Metformin synergistically enhanced cisplatin cytotoxicity and reversed chemoresistance by inhibiting the NF-κB/HIF-1α signaling axis, leading to the downregulation of hypoxia-regulated gene products.
Lin et al., 2014 [54], Taiwan	In vitro In vivo	Metformin/dasatinib	Cell culture/Cell viability assay/WB/Co-immunoprecipitation assay/Flow cytometry/Glucose measurement assay/ATP measurement assay/Live cell imaging/siRNA knockdown analysis/IHC staining and the scoring of p-AMPK and EGFR from HNSCC specimens/Subcutaneous ectopic xenograft tumor model	Ca9-22, HSC3, SAS	In vitro: Metformin: 0.5–10 mM; Dasatinib: 1–100 nM In vivo: Metformin: 250 mg/kg/day (oral); Dasatinib: 10 mg/kg/day (oral)	Well tolerated; no significant weight loss or toxicity observed; no damage to normal tissues	Metformin +/− dasatinib	Metformin sensitized dasatinib-induced anti-cancer effects through the activation of AMPK.

Abbreviations: TUNEL: Terminal deoxynucleotidyl transferase dUTP nick end labeling; WB: Western blot; qRT-PCR: Real-Time Quantitative Reverse Transcription PCR; IF: Immunofluorescence; CCK-8: Cell counting kit-8; EdU: 5-Ethynyl-2′-deoxyuridine; ELISA: Enzyme-linked immunosorbent assay; GSEA: gene set enrichment analysis; FACS: Fluorescence-activated cell sorting; HNSCC: Head and neck squamous cell carcinoma; PBS: Phosphate-buffered saline; 4SC-202: Domatinostat tosylate; OSCC: Oral Squamous Cell Carcinoma; CCL15: Chemokine ligand 15; TAM: Tumor-Associated Macrophage; SASP: senescence-associated secretory phenotype; M2-type: macrophage type 2; CCR1: C-C Motif Chemokine Receptor 1; CSC markers: Cancer Stem Cell Markers; 5-FU: Fluorouracil; AMPK: 5′ AMP-activated protein kinase; HIF-1α: Hypoxia-inducible factor 1-alpha; mTOR: mammalian target of rapamycin; LIN28: Lin-28 homolog A; SASP: Senescence-associated secretory phenotype; IL6: interleukin 6.

Only one study [56] explored the effects of the combination both in vitro and in vivo using C1632, a LIN28 inhibitor. This study showed that Metformin enhanced the anti-tumor effect of C1632 through amplified inhibition of the AMPK signaling pathway, suggesting a synergistic interaction.

Regarding the combination with curcumin, Metformin significantly enhanced curcumin’s pro-apoptotic and anti-proliferative effects in HPV-positive cell lines [49,57]. However, the study did not explicitly detail whether the interaction was synergistic or additive, nor did it quantify the extent of enhancement. Thus, while Metformin appears to potentiate curcumin’s known biological effects, the precise mode of interaction remains insufficiently described and requires further elucidation.

Across nine studies involving both in vitro and in vivo models, the co-administration of Metformin with various agents led to enhanced tumor control. For example, He et al. [12] demonstrated that Metformin combined with domatinostat tosylate (4SC-202), a selective class I HDAC inhibitor, significantly inhibited cell proliferation and promoted intrinsic apoptosis in OSCC both in vitro and in vivo.

In studies by Yin et al. [51,52], Metformin increased the effectiveness of the EGFR inhibitor gefitinib, enhancing tumor suppression in vitro and in vivo. The combination inhibited CCL15 expression in M2 tumor-associated macrophages and suppressed CCR1 expression on HNSCC cells, suggesting a cooperative immunomodulatory mechanism.

When combined with 5-fluorouracil (5-FU), Metformin enhanced anti-tumoral effects in OSCC cell lines by promoting cell growth inhibition and apoptosis through an independent pathway [24]. Similarly, Qi et al. [54] reported that Metformin augmented cisplatin cytotoxicity and reduced chemoresistance both in vitro and in vivo.

Further, in combination with curcumin, Metformin not only reduced tumor volume but also improved overall survival in treated models [53]. However, quantitative comparisons between monotherapies and combination therapy are not consistently presented, which limits interpretation of the strength of interaction.

Metformin was also shown to enhance Dasatinib-induced apoptosis via AMPK activation, promoting ER stress and EGFR degradation [59]. The combinatory inhibition of tumor progression was also observed with C1632, reinforcing the notion that Metformin may act as a metabolic sensitizer that enhances the effect of other targeted therapies [56].

### 3.4. Risk of Bias Across Studies

While the selected studies for this review were focused on head and neck cancer, they are highly heterogeneous in terms of reported data, study objectives, and outcome measures, making a meta-analysis unfeasible. Furthermore, several studies present a potential risk of bias due to limitations such as small sample sizes, lack of randomization or blinding, and inconsistent reporting of methodologies, which further undermines the comparability and overall reliability of the results.

## 4. Discussion

After sixty years of clinical experience and research, Metformin has exhibited no significant clinical adverse effects in diabetic patients. Clinical data suggest it may improve loco-regional tumor control, disease-free survival, and overall outcomes in head and neck tumors. While the clinical impact of Metformin in HNSCC is increasingly being demonstrated [60], the biological mechanisms underlying this anti-cancer effect are still not fully understood [61]. The two main proposed mechanisms involve an indirect effect through modulation of insulin resistance—by reducing insulin IGF1 (Insulin like Growth Factor) signaling and controlling hyperglycemia—as well as a direct effect on cell cycling and apoptosis. Hence, one may speculate that both indirect and direct mechanisms of Metformin may act in synergy.

The present systematic review provides the first comprehensive overview of in vitro and in vivo studies discussing the effects and the biological mechanisms of action of Metformin in HNSCC, updating the results published in 2017 by Rego et al.

The mTOR signaling pathway plays a key role in controlling cell metabolism, survival, growth, and proliferation. It is regulated, among others, by the IGF 1/PI3K/AKT signaling pathway, which is frequently dysregulated in human cancers. Several studies highlight that AMPK activation by Metformin downregulates the mTOR pathway through inhibition of mTOR complex 1 (mTORC1) in HNSCC. Madara et al. demonstrated anti-tumoral effects of Metformin, such as inhibition of mTOR signaling through expression of OCT3, a Metformin uptake transporter. Inhibition of OCT3 activity may interrupt AMPK activation and mTORC1 pathway inhibition. Metformin also inhibits mitochondrial complex I activity, which may lead to AMPK activation and mTOR inhibition. Inhibiting AMPK resulted in the loss of Metformin-induced AMPK activation and reduced initiation of apoptosis, highlighting the likely key role of AMPK in Metformin-induced apoptosis. YAP, a tumor promoter, enhances the proliferation rate of cells and reduces apoptosis in OSCC. Alteration of YAP by Metformin may provoke inhibition of mTOR and c Myc expression, suggesting a potential anti-tumoral effect by reducing cell proliferation and promoting apoptosis in OSCC. Studies in thyroid tumors suggest that Metformin increases p AMPK, reduces mTOR phosphorylation, downregulates S6K1/S6 signaling, and inhibits cyclin D1 and c MYC via the mTOR pathway. The analysis of these observations in the literature suggests a consistent phenomenon, even if the precise stages observed are different.

The effect of Metformin alone on proliferation has also been described in association with other drugs, especially with curcumin, 4SC 202, 5 FU, BPTES, or C1632. Those combinations may suppress tumor cell proliferation and may extend overall survival in animal xenograft models. Comparable effects are observed when combining Metformin with curcumin in prostate cancer cell lines [62].; this combination may lead to increased apoptosis, cytotoxicity, and expression of the Bax gene.

The combination of Metformin with 4SC 202, a selective class I histone deacetylase inhibitor, may suppress proliferation and promote intrinsic apoptosis of OSCC cells in vitro and in vivo through accelerated ubiquitin mediated degradation of ΔNp63. ΔNp63 is a homolog of p53 overexpressed in epithelial cancers and has oncogenic properties. Combined use with CDK4/6 inhibitors suggests Metformin may synergistically inhibit HNSCC by inducing cell cycle arrest. [58].

Another main effect attributed to Metformin is reported through regulation and arrest of the cell cycle. Cancer progression is caused by multiple genetic changes that induce dysregulation of the G1 to S transition. In prostate cancer, a similar effect was observed, showing Metformin’s capacity to reduce cell viability and to arrest at the G1 phase. During the cell cycle, the G1 phase is controlled by interactions among cyclins D and E, CDKs 2, 4, and 6, and CDK inhibitors. Sikka et al. showed that Metformin provoked a G1 arrest concomitant with reductions in protein levels of CDK2, 4, 6, cyclins D and E, and certain CDK inhibitors (p15, p16, p18, p27), but no change in others (p19 and p21), as well as de-creases in oncogenic proteins Skp2 and β TrCP, ultimately leading to arrest at the G0/G1 phase. These effects on the G0/G1 phase were also observed by Luo et al. in OSCC cells, although without effect on p21 and p27 expression.

Moreover, in vitro inhibition of cancer/metastasis development by Metformin via Late SV40 Factor (LSF)/Aurora A signaling has been reported. Metformin may decrease expression of Aurora A by blocking LSF, which is important in cell cycle progression, thereby impairing LSF/Aurora A signaling. Metformin also appears to exert anti-tumor effects by inhibiting proteolysis of NGFR, a transmembrane protein involved in cell proliferation and metastasis in prostate cancer and HNSCC.

In addition, Wu et al. observed sensitization of FaDu cells to Taxol and radiotherapy with Metformin treatment via modulation of IGF 1, leading to decreased SNHG7 expression. SNHG7 is a pro-oncogene that regulates the cell cycle by targeting GALNT1, cyclin D1, FAIM2, p15, and p16. Its expression level is a negative prognostic factor in terms of overall survival in patients with hypopharyngeal cancer.

Apoptosis induction is another major effect of Metformin treatment on HNSCC cells. Apoptosis is a key cellular mechanism controlling cell survival and death, with caspases playing a central role, active via extrinsic or intrinsic signals. In this context, Yang et al. showed that the association of the glutaminase inhibitor BPTES and Metformin may induce apoptosis by stimulating cleavage of pro-caspase 3 to its active form. An increase in caspase 3 transcription was also reported by Guimarães et al. A high Bax/Bcl 2 ratio is essential to favor the penetration of Bax into mitochondria and the subsequent release of cytochrome C. The intrinsic apoptosis pathway depends on cytochrome C release from mitochondria. In addition, Metformin-induced downregulation of anti-apoptotic proteins Bcl 2 and Bcl xL, and overexpression of the pro-apoptotic protein Bax. Similarly, Zhang et al. observed that Metformin may modulate the Bcl 2/Bax axis, further inducing apoptosome assembly and caspase 9 activation, leading to apoptosis. Also, Sun et al. demonstrated that in a dose and time dependent manner Metformin significantly inhibited FaDu cell proliferation and downregulated miR 21 5p while upregulating the mRNA and protein expression of its downstream target gene Programmed Cell Death 4, a pro-apoptotic tumor suppressor gene.

Furthermore, Metformin may foster tumor cell apoptosis through alterations of the immune tumor microenvironment.

All of these factors result in a decreased tumor proliferation rate. Patil demonstrated that the efficacy of Metformin in decreasing CD44 expression may depend on its concentration. Moreover, Metformin co-cultured with normal oral fibroblasts may initiate autophagy in OSCC. A senostatic effect of Metformin was also reported, showing its ability to modulate the profile of the senescence-associated secretory phenotype elicited by LY2835219, a CDK4/6 inhibitor in HNSCC, by inhibiting the mTOR and STAT3 pathways.

Metformin also shows metabolic effects on HNSCC. Under hypoxic conditions, it may promote anti-tumoral effects by increasing pyruvate dehydrogenase (PDH) levels and reducing hypoxia-inducible factor 1 alpha (HIF 1α) mRNA, a key regulator in adaptation to hypoxia. It appears to downregulate HIF 1α expression and inhibit activation of NF κB, which directly modulates HIF 1α under hypoxic conditions. Metformin also induces a significant reduction in MCT1, which acts as a lactate importer in cancer, thereby potentially impeding higher cancer cell proliferation through the reverse Warburg effect.

Notwithstanding, Metformin has garnered significant interest for its potential anti-tumor effects [63]. One retrospective study reported that, among diabetic patients with HNSCC of the larynx and oropharynx, those treated with Metformin exhibited significantly improved outcomes after a five year follow up: the Metformin group showed higher overall survival (72.7% vs. 34.7%, *p* < 0.038) and a much lower incidence of distant metastasis (18.1% vs. 82.6%, *p* < 0.001). However, it is important to note that the control group in that study was not clearly defined. It remains unclear whether these patients were also diabetic but untreated with Metformin, or whether they represented a broader HNSCC population irrespective of diabetic status. If the latter, the extremely high metastasis rate (82.6%) raises concerns and warrants cautious interpretation of the data.

Beyond HNSCC, Metformin also appears to improve prognosis in a range of other malignancies, including lung, colorectal, prostate, breast, kidney, and pancreatic cancers. Currently, several clinical trials are underway (Clinical-Trials.gov) to evaluate both its therapeutic and preventive potential. These include studies on chemoprevention in oral potentially malignant lesions (NCT03684707), premalignant lesions (NCT02581137, NCT05237960), and Metformin in combination with surgery plus either doxycycline (NCT03076281) or durvalumab (NCT03618654) [64,65,66,67,68]. However, while preliminary data are promising, clinical evidence supporting a survival benefit of Metformin in patients with HNSCC remains inconsistent and requires further investigation through well-designed prospective trials.

Limitations: The present review has several methodological limitations, the most significant being the near-total absence of quantitative data across the included studies. This gap limits the ability to identify the most promising drug combinations involving Metformin in terms of maximizing anti-tumor efficacy while minimizing adverse effects. A structured presentation of the available data—such as effective concentrations, IC_50_ values, and toxicity profiles—is essential to inform the rational use of Metformin in systemic or local combination therapies, as well as in chemoprevention or tertiary prevention strategies following primary treatment. Nevertheless, the review’s primary strength lies in its com-prehensive description of experimental studies evaluating Metformin’s role when combined with other agents. We also provide a detailed synthesis of numerous in vitro and in vivo investigations exploring Metformin’s anti-cancer mechanisms in head and neck squamous cell carcinoma.

## 5. Conclusions

Metformin has emerged as a clinically actionable adjuvant therapy in head and neck squamous cell carcinoma (HNSCC), offering a low-cost and well-tolerated option to enhance treatment efficacy. From a clinical standpoint, Metformin has demonstrated multiple benefits not only as a monotherapy but more notably as an adjunct to existing treatments. It has shown the ability to potentiate the effects of various chemotherapeutic agents, including gefitinib [51,52], BPTES [20], 4SC-202 [59], 5-fluorouracil (5-FU) [24], curcumin [53], and dasatinib [55]. Positive outcomes have also been reported with Taxol [29], although data on its combination with cisplatin remain somewhat inconsistent—yet overall suggest a beneficial adjuvant effect [50,54].

The mechanisms underlying these effects are diverse, primarily involving modulation of the mTOR signaling pathway, leading to reduced tumor cell proliferation, cell cycle arrest, enhanced apoptosis, metabolic reprogramming of the tumor microenvironment, suppression of immune tolerance, and improved cytotoxic immune responses.

These findings suggest that Metformin holds strong potential as a synergistic partner in combination regimens for HNSCC. Given its favorable safety profile and low cost, it prompts an important discussion on its potential off-label use in non-diabetic patients. Could HNSCC outcomes be significantly improved without increasing treatment-related toxicity? If so, should health insurance providers support its use based on a favorable cost–benefit ratio?

These questions are particularly relevant in the current context of value-based care and precision oncology. Addressing them will require further in vitro studies and well-designed clinical trials to validate these findings, identify optimal therapeutic combinations, and define the patient populations most likely to benefit

## Data Availability

The data is available at the Maxillofacial Surgery Secretariat at CHU Saint Pierre in Brussels, Belgium.
